# In Vivo Precision Evaluation of Lymphatic Function by SWIR Luminescence Imaging with PbS Quantum Dots

**DOI:** 10.1002/advs.202206579

**Published:** 2023-01-01

**Authors:** Xinxian Meng, Huizhu Li, Yuzhou Chen, Liman Sai, Sijia Feng, Ke Li, Wenjing Xi, Yunxia Li, Nguyen T. K. Thanh, Yueming Wang, Yan Wo, Xing Yang, Yuefeng Hao, Yixin Zhang, Jun Chen, Shaoqing Feng

**Affiliations:** ^1^ Department of Plastic and Reconstructive Surgery Shanghai Ninth People's Hospital School of Medicine Shanghai Jiao Tong University 639 Zhizaoju Rd. Shanghai 200011 P. R. China; ^2^ Sports Medicine Institute of Fudan University Department of Sports Medicine Huashan Hospital Fudan University Shanghai 200040 P. R. China; ^3^ Department of Physics Shanghai Normal University Guilin Road 100 Shanghai 200234 P. R. China; ^4^ Biophysics Group Department of Physics and Astronomy University College London Gower Street London WC1E 6BT UK; ^5^ UCL Healthcare Biomagnetic and Nanomaterials Laboratories 21 Albemarle Street London W1S 4BS UK; ^6^ Department of Anatomy and Physiology School of Medicine Shanghai Jiao Tong University Shanghai 200025 P. R. China; ^7^ Department of orthopedics Affiliated Suzhou Hospital of Nanjing Medical University Suzhou 215500 P. R. China

**Keywords:** lymphatic function, lymphatic system, lymphography, shortwave infrared (SWIR)

## Abstract

Advancements in lymphography technology are essential for comprehensive investigation of the lymphatic system and its function. Here, a shortwave infrared (SWIR) luminescence imaging of lymphatic vessels is proposed in both normal and lymphatic dysfunction in rat models with PbS quantum dots (PbS Qdots). The lymphography with PbS Qdots can clearly and rapidly demonstrate the normal lymphatic morphology in both the tail and hind limb. More importantly, compared to ICG, SWIR luminescence imaging with PbS Qdots can easily identify the dominant lymphatic vessel and node with higher luminescence signal in rats. Moreover, lymphatic pump is identified as segment contracting sections with a size of ≈1 cm in rat by in vivo SWIR lymphograhy, which propose a direct feature for precise evaluation of lymphatic function. Notably, in vivo SWIR luminescence imaging with PbS Qdots also clearly deciphers the in vivo pattern of morphological and function recovery from lymphatic system in rat model. In summary, SWIR luminescence imaging with PbS Qdots can improve the lymphography and thus deepen the understanding of the morphology and structure of the lymphatic system as well as lymphatic function such as lymphatic pump, which will facilitate the diagnosis of lymphatic dysfunction in the future.

## Graphical abstract

The shortwave infrared (SWIR) luminescence imaging with PbS quantum dots (PbS Qdots) is presented for the precise detection of lymphatic structure as well as the evaluation of lymphatic function represented by the lymphatic pump in both normal and pathological status, which will deepen the understanding of the lymphatic system and thus facilitate the diagnosis of lymphatic dysfunction in the future.

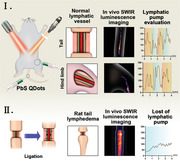

## 1 Introduction

Lymphatic system is a complex network mainly composed of lymphatic vessels and lymph nodes, which is essential for the maintenance of fluid homeostasis and immunocompetence of the body.^[^ ^1^ ^]^ Dysfunction of the lymphatic system leads to lymphedema, a troublesome disease that can result in recurrent episodes of skin infections, sepsis, and even disability.^[^ ^2^ ^]^ To date, however, knowledge of the network and function of the lymphatic system is still severely restricted.^[^ ^3^ ^]^ Lymphography, which assesses the lymphatic drainage and identifies the pathological status, is recognized as an imperative technique that aids improved understanding of lymphatic system.^[^ ^1^, ^4^ ^]^

Nowadays, current clinical lymphography technologies include lymphoscintigraphy, first near‐infrared (NIR, 700–900 nm) luminescence, and magnetic resonance imaging (MRI).^[^ ^5^ ^]^ Among them, NIR luminescence is a newly developed and highly attractive lymphography technique, and is mainly realized based on indocyanine green (ICG), an FDA‐approved NIR tracer.^[^ ^6^ ^]^ NIR luminescence lymphography with ICG presents real‐time dynamic visualization of lymphatic architecture delineation, which is hard to achieve with lymphoscintigraphy and MRI lymphography.^[^ ^5^ ^]^ However, NIR luminescence lymphography with ICG is stumbled by its inherent drawbacks such as limited detection depth and strong self‐luminescence background for NIR luminescence as well as poor stability and self‐quenching for ICG.^[^ ^6a^ ^]^ As a result, the spatial and temporal resolution of NIR luminescence lymphography with ICG is insufficient for detailed observation of lymphatic system. To date, it remains difficult by NIR luminescence lymphography with ICG to visualize superficial initial capillary lymphatic network, which is much smaller in diameter measuring around only 0.3 mm.^[^ ^7^ ^]^ Most importantly, direct and quick examinations of lymphatic function, represented by demonstrating the state of lymphatic pump, which is vital in the evaluation and dealing of lymphedema, is still not yet realized with NIR luminescence lymphography.^[^ ^8^ ^]^ Therefore, luminescence lymphography with higher imaging quality, with which functional evaluations can be approached, is urgently required for the evaluation of lymphatic function.

With less photon absorption and scattering by tissues, the shortwave infrared (SWIR) window(1,000−1,700 nm) luminescence imaging provides real‐time information that probes deep into the body with increased spatial and temporal resolution.^[^ ^9^ ^]^ Thus, SWIR imaging has emerged as a prospective technique for in vivo bioimaging.^[^ ^10^ ^]^ For example, it has been applied for the in vivo imaging of intricate structures such as vascular system.^[^ ^11^ ^]^ Moreover, our previous studies confirmed that lead sulfide quantum dots (PbS Qdots) products were a group of excellent SWIR image tracer for in vivo, real‐time and long‐time imaging of blood vessel and nervous system.^[^ ^12^ ^]^ Therefore, in vivo SWIR imaging with PbS Qdots is a promising candidate for lymphography demanding intricate visualization and functional assessments by providing high resolution and real‐time images.

**Scheme 1:**
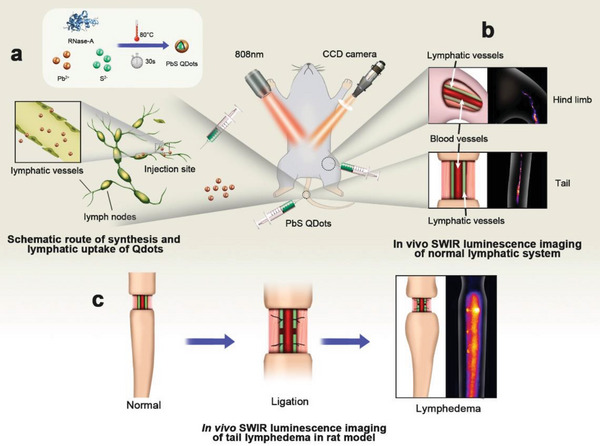
In vivo SWIR luminescence imaging of lymphatic system and lymphedema. a) Schematic route of synthesis and lymphatic uptake of PbS Qdots, respectively; In vivo SWIR luminescence imaging b) Normal lymphatic system and c) tail lymphedema in rat model.

Hence, in this study, SWIR luminescence imaging of the lymphatic system with PbS Qdots was investigated in rat models (**Scheme** **1**). Highly fluorescent PbS Qdots was synthesized and injected intradermally to perform the imaging of lymphatic system (Scheme 1a). Both normal and dysfunctional lymphatic system was performed for comparing and identifying their features with in vivo SWIR luminescence imaging. First, both tail and hind limb of normal rats were explored by image tracer injection (Scheme 1b). While rat tail lymphedema model was achieved and further investigated by in vivo SWIR luminescence imaging (Scheme 1c). Then biosafety for lymphography using PbS Qdots was confirmed with histopathological analysis and luminescence imaging of major organs. This work will propose an effective imaging method for demonstrating precise structures of the lymphatic system with high resolution and deep penetration, thus providing characteristic parameters for future functional evaluation for lymphatic system.

## 2 Results and Discussion

### 2.1 The Characterization of Lymphatic Tracer

**Figure 1:**
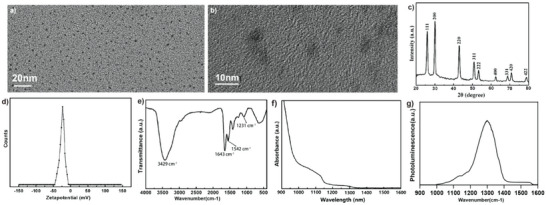
Characterization of PbS Qdots tracer. a) TEM images, b) HRTEM image of the PbS Qdots. c) XRD pattern and d) Zeta‐potential measurement of the PbS Qdots e) FTIR spectrum of the PbS Qdots. f) UV–vis and g) photoluminescence (PL) spectra of the PbS Qdots

PbS Qdots with SWIR fluorescent emission were directly synthesized in water by using RNase A enzyme as the ligand according to our previously reported methods.^[^ ^12a^ ^]^ Typical transmission electron microscopy (TEM) images are shown in **Figure** **1**a,b. The PbS Qdots showed near‐spherical structure with good monodispersity with size of ≈5 nm, which was in agreement with our previous results from TEM.^[^ ^12d^ ^]^ Figure 1c,d shows the XRD and zeta potential of the PbS Qdots, respectively. In Figure 1d, the diffraction peaks could be assigned to crystal planes of face‐center‐cubic rocksalt structured PbS (JCPDS No. 005–0592). The sharp peaks indicated well crystallization of the Qdots. No impurities were found, revealing their high purity. As shown in Figure 1e, the PbS Qdots had a surface potential of −22.8 mV, which could result from negatively charged chemical groups in RNase‐A ligand such as carboxyl or hydroxyl.

Additionally, FTIR and XPS spectroscopy were used to investigate the chemical structure of the PbS Qdots. In Figure 1e, O—H/N—H stretching vibration was found at 3,429 cm^−1^. Peaks at 1,643 and 1,542 cm^−1^ could be assigned to C=O and N—H groups in amides. In the XPS spectrum (Figure S1b, Supporting Information), a large amount of N, O, and C could be found, which belonged to the RNase‐A protein. The amplified picture showed the existence of Pb and S. The peaks at 136.4 and 141.1 eV corresponded to the binding energies of Pb 4f7/2 and Pb 4f5/2 core levels, indicating only one chemical phase. The peak at 163.1 eV belonged to binding energy of S 2p1/2, which was consistent with the previous report.^[^ ^13^ ^]^

Finally, the optical property of the PbS Qdots was characterized by their UV–vis and PL spectra. As shown in Figure 1f,g, the absorption of the PbS Q dots exceeded 1,000 nm, and a strong PL peak at 1,300 nm was observed upon 768 nm excitation, showing its excellent SWIR luminescence.

### 2.2 In Vivo SWIR Luminescence Imaging of Normal Lymphatic Vessels in Rat Tail

**Figure 2:**
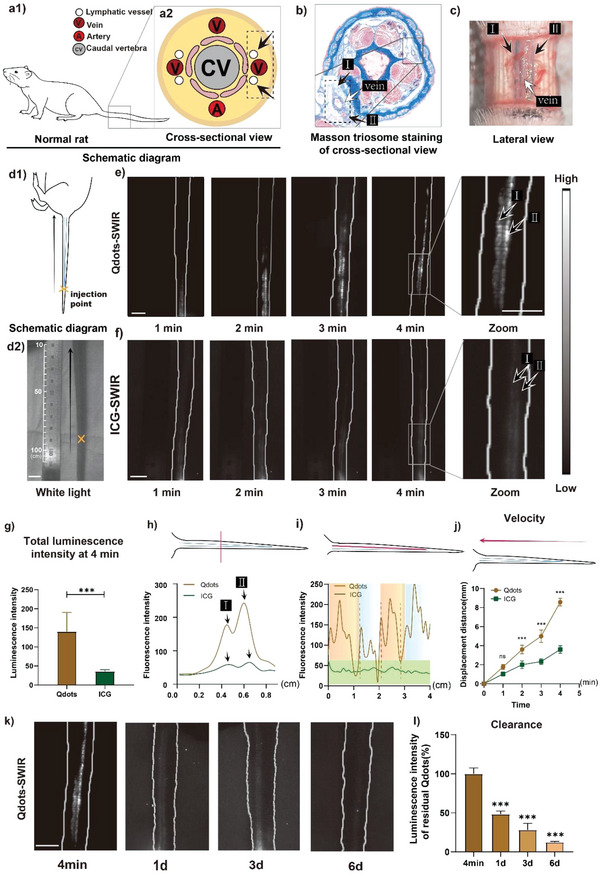
SWIR luminescence imaging of normal lymphatic vessel of rat tail. a): Scheme of anatomical position of lymphatic vessels of the SD rat in the cross‐sectional view. V: vein, A: artery, CV: caudal vertebra. The lymphatic vessels are indicated with black arrows. b) The Masson trichrome staining indicating the anatomical position of lymphatic vessels. c) Picture of tail lymphatic vessels on one side of the tail after the excision of circumferential full‐thickness skin and the injection of methylene blue. The lymphatic vessels were labeled with black arrows. d) d1: Schematic illustration and d2: White light pictures showing the injection point (orange cross) and the direction of lymphatic drainage (black arrow, scale bar: 1 cm). e) and f) SWIR luminescence imaging of normal lymphatic vessels on one side of the tail at 1, 2, 3, and 4 min after the injection of PbS Qdots and ICG, respectively. Lymphatic vessels were labeled with arrows in the zoomed image. Scale bar: 1 cm. g) Quantitative analysis of luminescence intensity in (f) with PbS Qdots and ICG at 4 min, respectively. Quantitative analysis of luminescence intensity of h) the cross‐sectional level and (i) the longitudinal axis of rat tail lymphatic vessels of PbS Qdots and ICG. j) The infusion velocity within 4 min post‐injection k) and l) the images acquired 4 min and 1, 3, 6 d after injection and quantitative analysis. Scale bar: 1 cm. (*n* = 3) Data are represented as the mean ± SD, with ^*^ *p* < 0.05, ^**^ *p* < 0.01, and ^***^ *p* < 0.001.

To begin with, normal lymphatic system in the rat tail was first studied (**Figure** **2**). As shown in Figure 2a–c, the anatomical position of lymphatic vessels in rat tail was explored. Masson trichrome staining demonstrated that there were four lymphatic vessels in the rat tail accompanying the veins (Figure 2b). Furthermore, circumferential removal of full‐thickness skin of the tail was performed to assist in recognition of lymphatic vessels with methylene blue (MB) in the lateral view. As the lymphatic vessels located laterally in the tail, only two blue‐colored lymphatic vessels with MB filling were observed in the lateral view, as shown in Figure 2c. Since following in vivo SWIR imaging of the tail would be realized in the lateral view, only two of the four lymphatic vessels were to be presented in images.

Subsequently, in vivo SWIR luminescence imaging was performed. Twenty microliters as‐prepared PbS Qdots was administered intradermally at the end of the rat tail (Figure 2d). As shown in Figure 2e and Video S1 (Supporting Information), the PbS Qdots rapidly infused into and filled the lymphatic vessels along the rat tail from the injection point toward the proximal portions of the rat tail after 4 min post injection. Meanwhile, an excision of circumferential full‐thickness skin was conducted to further verify whether the luminescence was from the lymphatic vessels instead of blood vessels. Two lymphatic vessels with luminescence signals could be clearly observed within the excision site (Figure S2, Supporting Information), confirming the successful SWIR lymphography achieved by PbS Qdots. On the other hand, rats that underwent injections of the same amount of ICG to the same location of the tail served as controls in this live animal imaging. As shown in Figure 2f (Video S2, Supporting Information), the outline of two lymphatic vessels in the ICG group was not as clear as PbS Qdots group. Therefore, in SWIR luminescence imaging, the PbS Qdots, as a lymphatic tracer, can easily identify the morphology of lymphatic vessels with better imaging quality in comparison with ICG.

Meanwhile, corresponding statistical analysis revealed that the luminescence intensity of the PbS Qdots group was significantly higher than that of the ICG group (Figure 2g). Besides, in the analysis of the cross‐sectional luminescence intensity of the rat tail, two signal peaks that matched the position of two lymphatic vessels are presented in Figure 2h. Furthermore, the two peaks of the PbS Qdots group were more obvious than the ICG group, indicating that the difference between the signal of lymphatic vessels and background was more significant. Notably, the peak I was weaker than the peak II in PbS Qdots group throughout the observation (Figure 2e,h). Meanwhile, the vessel with higher signal intensity presented significantly higher transportation velocity (Figure 2e,f,h; Videos S1 and S2, Supporting Information). It might ascribe to the peak II as the dominant lymphatic vessels in lymphography. Owing to the existing preferential lymphatic drainage pattern, the dominant lymphatic vessel would have higher luminescence signal intensity and transportation velocity.^[^ ^14^ ^]^ Nevertheless, such a difference was not detected in images of the ICG group (Figure 2f,h). Consequently, comparing with ICG, PbS Qdots would be a better lymphatic tracer in the SWIR window, which enabled a convenient recognition of the lymphatic vessels with more detailed structure.

Additionally, function of lymphatic vessels could be assessed directly by the state of the lymphatic pump. In lymphography, the drainage of lymphatic fluid is driven by segmental contractions of lymphatic vessels, which are termed as the lymphatic pump. In Figure 2e, the luminescence signal intensity along the lymphatic vessels in these images of the PbS Qdots group clearly presented a segmental pattern, which was ascribed to the existence of the lymphatic pump. By analysis of the distribution of signal intensity along the vessels, lymphatic pump was easily identified as there were series of segmental contracting sections, and the size of lymphatic pump in rat tail lymphatic vessels was estimated to be ≈1 cm (marked by the color blocks in Figure 2i). Distribution of signal intensity on the images of the ICG group, however, provided less information owing to low luminescence intensity and sensitivity, with which contracting sections of lymphatic vessels were undistinguishable. Therefore, in vivo SWIR imaging with PbS Qdots showed its superiority in providing more details in decoding the characteristics of the lymphatic pump in comparison with the ICG group, meeting growing demands for functional assessments in lymphography.

Finally, knowledge of the cleanout of tracer is imperative for future biomedical application. Figure 2j,i describes the characteristics of cleanout of PbS Qdots when being administrated for lymphography of rat tails. As shown in Figure 2j, the infusion velocity of ICG in lymphatic vessels (0.1 mm s^−1^) was significantly slower than that of PbS Qdots (0.35 mm s^−1^), indicating that the lymphography with PbS Qdots could be finished in a shorter time. Furthermore, the luminescence in lymphatic vessels of rat tail faded within 6 d in the rat (Figure 2k,l), which was fundamentally shorter than the retention time of ICG that was reported to be longer than 14 d.^[^ ^15^ ^]^ Rapid cleanout of PbS Qdots makes it possible to shorten the interval between repetitive examinations.

### 2.3 In Vivo SWIR Luminescence Imaging of Normal Lymphatic Vessel in Rat Hind Limb

**Figure 3:**
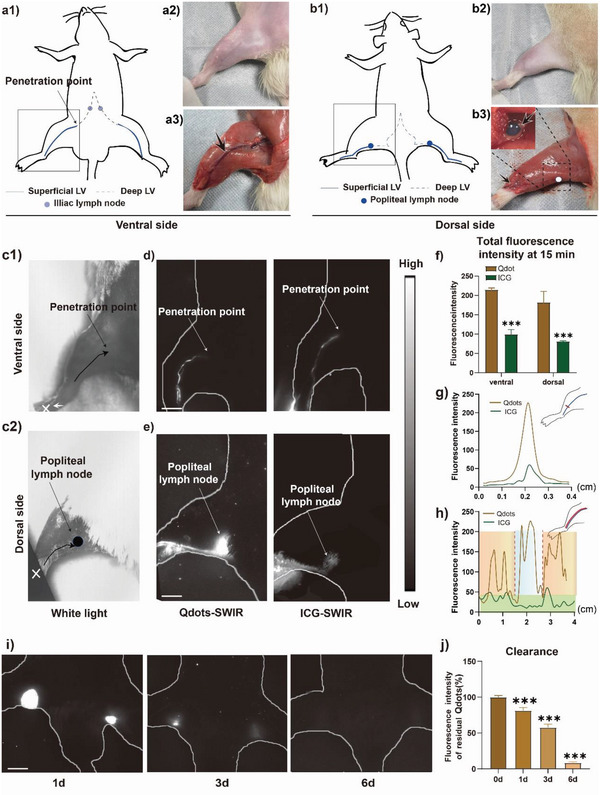
In vivo SWIR luminescence imaging of normal lymphatic vessel of rat hind limb. Scheme of the lymphatic vessels on (a1) the dorsal and (b1) the ventral side of the rat. a2 – 3 and b2 – 3 Picture of corresponding lymphatic vessels after removal of the skin and the injection of methylene blue. The lymphatic vessels were labeled with black arrows. c) White light picture showing the injection point (white point pointed with the white arrow) and the direction of lymphatic drainage (black arrow). d,e) SWIR luminescence imaging of normal lymphatic vessels on one side of the hind limb 15 min after the injection of PbS Qdots and ICG. On the ventral side, lymphatic vessels were found along the great saphenous vein and then penetrated deeper near the beginning of the thigh. On the dorsal side of the hind limb, a pair of lymphatic vessels was found along the short saphenous vein, penetrating the biceps femoralis and leading to the popliteal lymph node underneath. Scale bar: 1 cm. f) Quantitative analysis of images acquired with PbS Qdots and ICG on dorsal and ventral side, respectively. g) Quantitative analysis of luminescence intensity along the cross‐sectional level of the rat tail lymphatic vessels of PbS Qdots and ICG. h) The distribution of signal intensity manifesting the lymphatic pump along the longitudinal axis of lymphatic vessels with PbS Qdots and ICG on the ventral side. i,j) the images acquired after 1, 3, and 6 d after injection and the quantitative analysis of the luminescence intensity. Scale bar: 1 cm. (*n* = 3) Data are represented as the mean ± SD, with ^*^ *p* < 0.05, ^**^ *p* < 0.01, and ^***^ *p* < 0.001.

To further confirm the practicability of PbS Qdots for lymphography, in vivo SWIR luminescence imaging of the lymphatic vessels of rat hind limbs was repeated as they are morphologically different from the lymphatic system of tails (**Figure** **3**a1,b1). As shown in Figure 3a2 and b2, the outline of lymphatic vessels of rat hind limb could be barely recognized with filling of MB after shaving the skin. Figure 3a2 and b2 demonstrates that the lymphatic vessels of hind limb were discovered to be located both on the dorsal and the ventral side along the artery and vein after removal of the superficial tissues.

Afterward, in vivo SWIR luminescence imaging with PbS Qdots and ICG was performed, respectively. The morphology of lymphatic vessels was presented in both the PbS Qdots group and the ICG group, and the locations were in accordance with the anatomical results (Figure 3e). Nevertheless, the images of the PbS Qdots group demonstrated clearer structures (Figure 3e) with remarkably higher total luminescence intensity (Figure 3f) and more distinct peak of lymphatic vessels (Figure 3g). Notably, the segmental contracting section with ≈1 cm driven by the lymphatic pump was again visualized in images of the PbS Qdots group (Figure 3h; Videos S3 and S4, Supporting Information). On the contrary, the distribution of segmental contracting sections along the vessel in images of the ICG group was irregular with no recognizable pattern (Figure 3h), suggesting that ICG failed to present state of the lymphatic pump in the SWIR region. Hence, a direct representation of the lymphatic pump in rat hind limb was achieved but only with SWIR imaging based on PbS Qdots.

Concurrently, a dominant one could also be determined among two popliteal lymph nodes on the images of the PbS Qdots group. It is shown in Figure 3i that the signal intensity of the right popliteal lymph node was fundamentally weaker than the left one (Figure 3i). Likewise, it possibly resulted from the preferential lymphatic drainage.^[^ ^14^ ^]^ Furthermore, the luminescence in hind limb lymphatic vessels and lymphatic nodes of the PbS Qdots group faded within 6 d (Figure 3i,j) in agreement with the aforementioned results of rat tail.

To sum up, in vivo SWIR luminescence imaging based on PbS Qdots was utilized successfully to display the morphology of lymphatic vessels and the state of the lymphatic pump in lymphatic vessels of the tail and the hind limb of rats, indicating its potential for accurate evaluation of lymphatic function.

### 2.4 In Vivo SWIR Luminescence Imaging of Lymphatic Dysfunction

**Figure 4:**
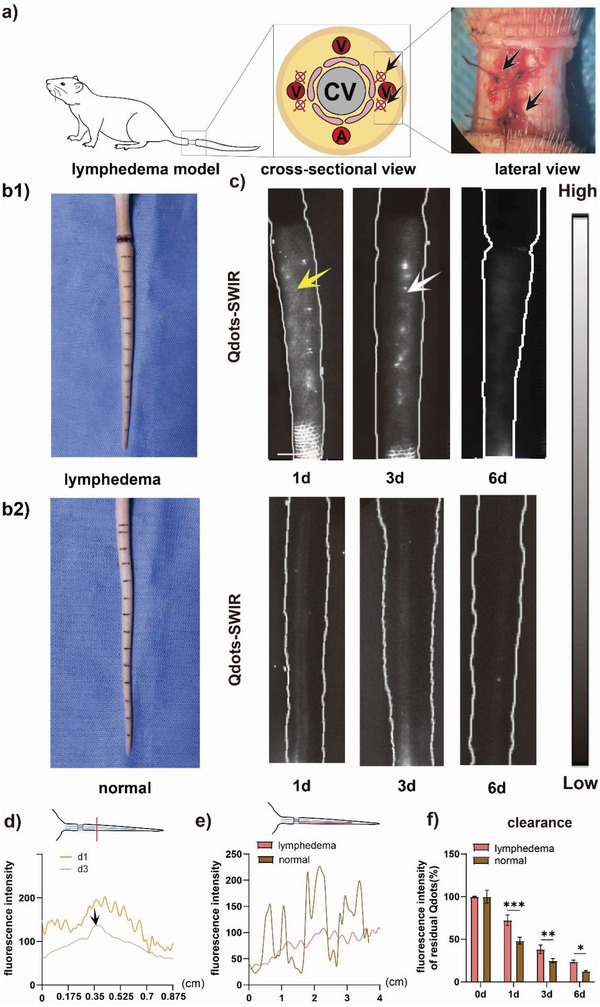
In vivo SWIR luminescence imaging with PbS Qdots of lymphatic dysfunction (lymphedema). a) Schematic illustration of rat tail lymphedema model. b1) Photo of normal rat tail b2) photo of lymphedema rat tail. c) In vivo PbS Qdots imaging of lymphedema rat tail and normal rat tail. Yellow arrow: dermal backflow, white arrow: lymphatic vessels. Scale bar: 1 cm. d) The cross‐sectional luminescence intensity of the swollen rat tail. e) The cross‐sectional luminescence intensity of the rat tail lymphatic vessels by PbS Qdots with or without lymphedema. f) The quantitative analyses of the images acquired 0, 1, 3, 6, and 9 d after injection. (*n* = 3) Data are represented as the mean ± SD, with ^*^ *p* < 0.05, ^**^ *p* < 0.01, and ^***^ *p* < 0.001.

Additionally, in vivo SWIR luminescence imaging of lymphatic dysfunction was also investigated to distinct its pathological and functional features from natural lymphatic system. A post‐surgical lymphedema model of the rat tail was established (**Figure** **4**a) and was comparatively analyzed by in vivo SWIR luminescence imaging (Figure 4b).

As shown in Figure 4b1, 2 weeks after surgery, the tail became swollen and the skin of the tail became hard and fibrotic, signaling that lymphedema was successfully induced in the tail of rat. Subsequently, 2 min after injection, PbS Qdots spread immediately to the peripheral region of the injection point in 0 d, displaying no structure of lymphatic vessels (Figure S3, Supporting Information). The diffusion of the tracer might be attributed to the abnormal accumulation of lymphatic fluid in dermal layer of the tail and could be taken as a sign of severely interrupted lymphatic drainage. As can be seen in Figure 4d, with the onset of lymphedema the pattern of lymphatic pump was also lost on SWIR images, directly reflecting lymphatic dysfunction. Still, on the 1 d post‐injection, scattered luminescence signal across the tail was discovered without normal structure of lymphatic vessels, which was probably due to severe dermal backflow (Figure 4c, yellow arrow), signaling persistent serious lymphatic dysfunction. Interestingly, scattered luminescence signal diminished slightly and the outline of lymphatic vessels emerged after 3 d of injection (Figure 4c, white arrow), but it remained hard to identify the two vessels, suggesting ongoing recovery of lymphatic function.

Besides, as shown in Figure 4d, distinctions of signal intensity between lymphatic vessels and peripheral region manifested over time and were recognizable on 3 d post‐injection, demonstrating that lymphatic vessels gradually became identifiable when lymphatic dysfunction was relieving. As shown in Figure 4f, the clearance of PbS Qdots in the rat tail with lymphatic dysfunction was significantly slower than that of the normal tail, evidencing hindered lymphatic drainage with lymphedema.

Generally, then in vivo SWIR luminescence imaging with PbS Qdots in lymphedema model proved to be sensitive to feature differences between normal and malfunctioned lymphatic vessels, and clearly deciphered the in vivo pattern of morphological recovery from lymphatic dysfunction in rat models of tail lymphedema. Therefore, this lymphography technique may help identify the underlying defected status of lymphatic system, suggesting great potential in diagnosis of lymphatic dysfunction.

### 2.5 The Biosafety of PbS Qdots for Lymphography

**Figure 5:**
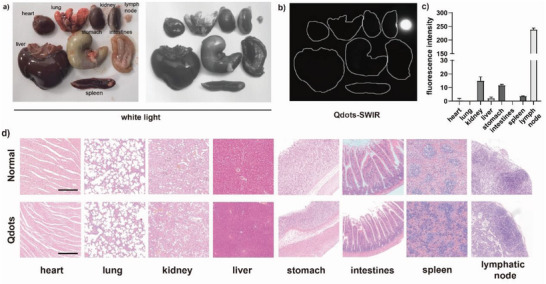
The biosafety assessment of PbS Qdots for lymphography. a) Ex vivo photo of major organs harvested from the rat treated with PbS Qdots. b,c) Ex vivo SWIR imaging of major organs harvested from the rat treated with PbS Qdots and the quantitative analysis. d) Representative histological H&E staining of major organ tissues harvested from the rat treated with sterile saline (normal) and PbS Qdots, Scale bar: 400 µm. (*n* = 3) Data are represented as the mean ± SD, with ^*^ *p* < 0.05, ^**^ *p* < 0.01, and ^***^ *p* < 0.001.

To investigate the potential toxicity of PbS Qdots as a lymphatic tracer, the PbS Qdots retention and histological analysis of organs are examined in **Figure** **5**. As shown in Figure 5a–c, no luminescence signal was detected in most of the organs from rat after 6 d post‐injection, except for the lymph nodes, indicating that PbS Qdots were mainly uptaken and metabolized by lymphatic system rather than other organs. Despite the luminescence signal in lymphatic system was hardly detected in vivo 6 d post‐injection, luminescence signal of lymph nodes ex vivo could be detected (Figure 5b,c). It was deduced that most of the injected PbS Qdots in the lymphatic system could be cleared out with 6 d, while a few of the PbS Qdots deposited in the lymph nodes for a longer time. As shown in Figure 5d, the results of H&E staining indicated that there were no notable inflammation or other abnormalities in major organs 6 d post‐injection as compared with normal organs. In a word, PbS Qdots is able to mainly target lymphatic system with good biosafety after injection, which could be a good lymphatic tracer for lymphography.

## 3 Conclusion

In summary, in comparison with ICG the in vivo SWIR luminescence imaging with PbS Qdots can more clearly demonstrate the morphology of lymphatic vessels and nodes in both tail and hind limb of rat. Besides, many lymphatic structures including dominant lymphatic vessel as well as lymphatic pump could only be detected by PbS Qdots injection. Notably, with direct imaging of the lymphatic pump in normal and lymphedema environment, PbS Qdots as a promising lymphatic tracer could identify the status of lymphatic dysfunction based on real‐time, high resolution, and long‐time SWIR luminescence imaging. Thereby, it was strongly believed that SWIR luminescence lymphography hopefully helps to evaluate the lymphatic function, which would play an important role in improvement of diagnosis of lymphatic diseases in the future.

## 4 Experimental Section

### Reagents

All chemicals were used without further purification. Ribonuclease A (RNase A) from bovine pancreas (MW: 13.7 kDa, >70 U mg^−1^), lead acetate trihydrate (Pb(OAc)_2_·3H_2_O, ≥99.9%), sodium sulfide nonahydrate (Na_2_S·9H_2_O, ≥98.0%), sodium hydroxide (NaOH, ≥98.0%), and ICG were purchased from Sigma–Aldrich.

### Animals

Sprague–Dawley (SD) rats (150‐200 g, male) were provided by the Animal Care Facility of School of Medicine Shanghai Jiao Tong University (Shanghai, China). All animal experiments were strictly performed under the guidelines of the Chinese Council for Animal Care, approved by the Animal Care Committee of the Laboratory Animal at School of Medicine, Shanghai Jiao Tong University.

### Synthesis of PbS Qdots

The synthesis of PbS Qdots was based on the previous report by Kong and Chen.^[^ ^16^ ^]^ Briefly, 500 µL of 50 mg mL^−1^ RNase‐A was mixed with 500 µL of 10 mm Pb(OAc)_2_, then 1 m NaOH solution was added to adjust the system pH to 9–11. After stirring, 50 µL of 10 mm Na_2_S aqueous solution was added. The mixture was reacted at 70 °C for 30 s in a microwave reactor with an input power of 30 W. The as‐prepared RNase‐A‐encapsulated PbS Qdots were dialyzed once (to remove Pb^2+^, OAc^−^, Na^+^, OH^−^, etc.), and washed with deionized water to neutralize system pH until it was close to neutral. At last, the washed PbS Qdots were centrifuged for concentration at a speed of 4,000 rpm speed centrifugation for 10 min at 4 °C. The freshly prepared PbS Qdots solution was dissolved in PBS solution and stored at 4 °C.

### Characteristics of PbS QDs

TEM and HR‐TEM imaging was conducted on a JEOL JEM‐2010F field emission TEM operated at 200 kV. The photoluminescence spectra of PbS Qdots were collected on an Applied NanoFluorescence spectrometer at room temperature with an excitation laser source of 685 nm. The FTIR spectrum was recorded on a Bruker Tensor II FTIR spectrophotometer (frequency range from 4,000 to 400 cm^−1^, Shanghai, China). The XPS analysis was carried out on a VG ESCALAB 220i‐XL (Beijing, China) surface analysis system. The UV–vis absorption spectrum of PbS Qdots was measured by a UV–vis spectrometer, background‐corrected for contribution from water. The measured range was from 900 to 1,600 nm. Zeta potential and size analysis were detected for dynamic light scattering analysis with a Zetasizer analyzer (Malvern Panalytical).

### Creation of Secondary Lymphedema in a Rat Tail Model

Post‐surgical lymphedema was induced in the tails of SD rats. The rats were anesthetized with sodium pentobarbital (50 mg kg^−1^ intraperitoneally). Next, 0.1 mL of 2% methylene blue solution was injected intradermally into the end of the rat tail. An annular incision was made at 13 cm from the end of the tail. The skin and subcutaneous tissue were removed, thus removing the superficial lymphatic network. The muscles, tendons, bones, and major blood vessels below the dermis were left untouched. A surgical microscope (Olympus, Japan) was used to visualize the two deep lymphatic vessels stained with methylene blue, and the vessels were sutured with 6–0 silk thread and cut. This study was approved by the Animal Ethics Committee of China. All methods were performed in accordance with the relevant guidelines and regulations.

### In Vivo SWIR Luminescence Imaging

The model rats were prepared for imaging after complete anesthesia. Twenty microliters of PbS Qdots was intradermally injected into the tail. The excitation light source was provided by an 808 nm diode laser. The light emitted from the rat was filtered by a 1,250 nm long‐pass filter, and SWIR images were obtained immediately with a 2D InGaAs camera (Photonic Science). At last, the rats were sacrificed to harvest their major organs and lymph nodes for ex vivo NIR‐II imaging and H&E stain.

### Luminescence Intensity, Lymphatic Pump Function, and Infusion Velocity Measurement

The luminescence intensity was measured by software ImageJ. The cross‐sectional luminescence intensity of the rat tail lymphatic vessels was analyzed by measuring the luminescence intensity along the axis perpendicular to the rat tail and the tail lymphatic vessels. The lymphatic pump function was analyzed by measuring the luminescence intensity along the lymphatic vessels. The infusion velocity was calculated by dividing the displacement distance from the injection point and the acquisition time.

### Statistical Analysis

All statistical analysis was performed using unpaired two‐tailed Student's *t*‐tests. Mean values ± SD were used to represent the data. *p* values are indicated as follows: ^*^ *p* < 0.05, ^**^ *p* < 0.01, and ^***^ *p* < 0.001. Quantification of the luminescence was using ImageJ. Pseudo‐color was adding by ImageJ.

## Conflict of Interest

The authors declare no conflict of interest.

## Supporting information

Supporting Information

Supplemental Video 1

Supplemental Video 2

Supplemental Video 3

Supplemental Video 4

## Data Availability

The data that support the findings of this study are available from the corresponding author upon reasonable request.

## References

[1] A. K. Polomska , S. T. Proulx , Adv. Drug Deliv. Rev. 2021, 170, 294. 32891679 10.1016/j.addr.2020.08.013

[2] a) S. G. Rockson , V. Keeley , S. Kilbreath , A. Szuba , A. Towers , Nat. Rev. Dis. Primers 2019, 5, 22; 30923312 10.1038/s41572-019-0072-5

[3] a) D. W. Chang , J. Masia , R. Garza , R. Skoracki , P. C. Neligan , Plast. Reconstr. Surg. 2016, 138, 209S; 27556764 10.1097/PRS.0000000000002683

[4] L. L. Munn , T. P. Padera , Microvasc. Res. 2014, 96, 55. 24956510 10.1016/j.mvr.2014.06.006 PMC4268344

[5] G. Kim , M. P. Smith , K. J. Donohoe , A. R. Johnson , D. Singhal , L. L. Tsai , Eur. Radiol. 2020, 30, 4686. 32221682 10.1007/s00330-020-06790-0

[6] a) H. Hara , M. Mihara , Y. Seki , T. Todokoro , T. Iida , I. Koshima , Plast. Reconstr. Surg. 2013, 132, 1612; 24005372 10.1097/PRS.0b013e3182a97edc

[7] a) W. R. Pan , C. M. le Roux , S. M. Levy , C. A. Briggs , Clin. Anat. 2010, 23, 654; 20533512 10.1002/ca.21004

[8] J. P. Scallan , S. D. Zawieja , J. A. Castorena‐Gonzalez , M. J. Davis , J. Physiol. 2016, 594, 5749. 27219461 10.1113/JP272088 PMC5063934

[9] S. Zhu , R. Tian , A. L. Antaris , X. Chen , H. Dai , Adv. Mater. 2019, 31, 1900321. 10.1002/adma.201900321 PMC6555689 31025403

[10] a) X. Meng , X. Pang , K. Zhang , C. Gong , J. Yang , H. Dong , X. Zhang , Small 2022, 18, 2202035; 10.1002/smll.202202035 35762403

[11] a) C. Li , G. Chen , Y. Zhang , F. Wu , Q. Wang , J. Am. Chem. Soc. 2020, 142, 14789; 32786771 10.1021/jacs.0c07022

[12] a) M. Chen , S. Feng , Y. Yang , Y. Li , J. Zhang , S. Chen , J. Chen , Nano Res. 2020, 13, 3123;

[13] J. Bu , C. Nie , J. Liang , L. Sun , Z. Xie , Q. Wu , C. Lin , Nanotechnology 2011, 22, 125602. 21317496 10.1088/0957-4484/22/12/125602

[14] M. Weiler , J. B. Dixon , Front Physiol. 2013, 4, 215. 23966950 10.3389/fphys.2013.00215 PMC3744037

[15] a) C. Hirche , H. Engel , L. Kolios , J. Cognie , M. Hünerbein , M. Lehnhardt , T. Kremer , Surg. Innov. 2013, 20, 516; 23275469 10.1177/1553350612468962

[16] Y. Kong , J. Chen , H. Fang , G. Heath , Y. Wo , W. Wang , Y. Li , Y. Guo , S. D. Evans , S. Chen , D. Zhou , Chem. Mater. 2016, 28, 3041. 27212793 10.1021/acs.chemmater.6b00208 PMC4869608

