# Supplementary material for: In Vivo Precision Evaluation of Lymphatic Function by SWIR Luminescence Imaging with PbS Quantum Dots
Source: Adv Sci (Weinh). 2023 Jan 1;10(7):2206579. doi: 10.1002/advs.202206579 (PMC9982568; doi:10.1002/advs.202206579)
Supplement: Supplementary file 1 — Supporting Information [file ADVS-10-2206579-s003.pdf]

***In vivo* fluorescence imaging of lymphatic system and lymphatic dysfunction by SWIR PbS Quatum Dots**

Xinxian Meng[1] , Huizhu Li[2] , Yuzhou Chen[2] , Yixin Zhang[1*] , Jun Chen[2*] , Shaoqing Feng[1*]

1. Department of Plastic and Reconstructive Surgery, Shanghai Ninth People's Hospital, School of Medicine, Shanghai Jiao Tong University, 639 Zhizaoju Rd., Shanghai 200011, P.R. China
2. Sports Medicine Institute of Fudan University, Department of Sports Medicine, Huashan Hospital, Fudan University, Shanghai 200040, P.R. China.

*Corresponding Author:

Yixin Zhang

Department of Plastic and Reconstructive Surgery, Shanghai Ninth People's Hospital, School of Medicine, Shanghai Jiao Tong University, 639 Zhizaoju Rd., Shanghai 200011, P.R. China

Email address: zhangyixin6688@163.com

Jun Chen

Sports Medicine Institute of Fudan University, Department of Sports Medicine, Huashan Hospital, Fudan University, Shanghai 200040, P.R. China.

Email address: biochenjun@fudan.edu.cn

Shaoqing Feng

Department of Plastic and Reconstructive Surgery, Shanghai Ninth People's Hospital, School of Medicine, Shanghai Jiao Tong University, 639 Zhizaoju Rd., Shanghai 200011, P.R. China

Email address: fmmufsq@163.com

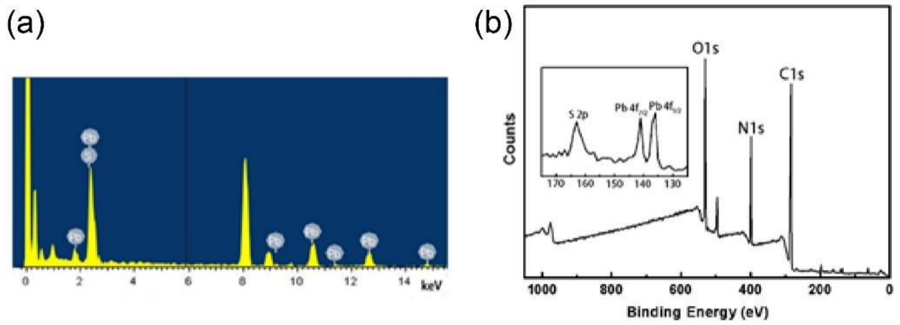

Figure S1 Characterization of PbS QDs (a) EDX spectrum of the PbS Qdots; (b) XPS survey spectrum of the PbS Qdots

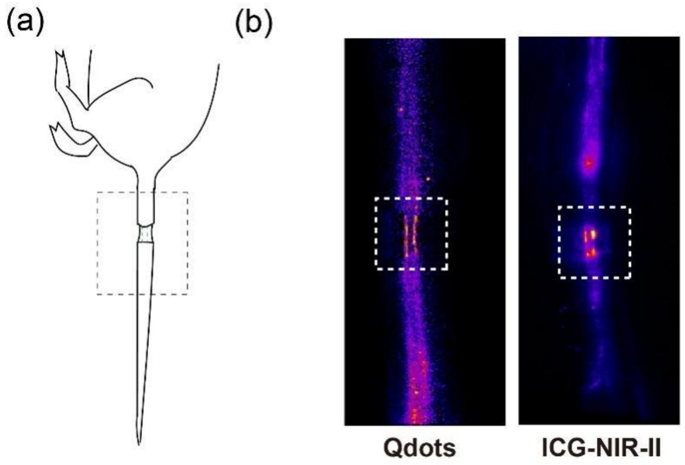

Figure S2 SWIR imaging of lymphatic vessels with skin peeled. a) schematic illustration. B) imaging with Qdots and ICG. the skin was peeled in the region within dotted box.

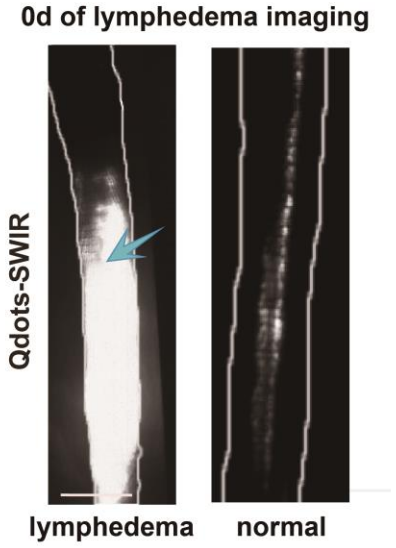

Figure S3 SWIR imaging of **luminescence imaging with PbS Qdots of lymphatic dysfunction (lymphedema) in day0 and of normal rat tail.** In vivo PbS Qdots imaging of lymphedema rat tail and normal rat tail. Blue arrow showed the immediate diffusion of the tracer, indicating the abnormal accumulation of lymphatic fluid in dermal layer of the tail. Scale bar: 1 cm.
